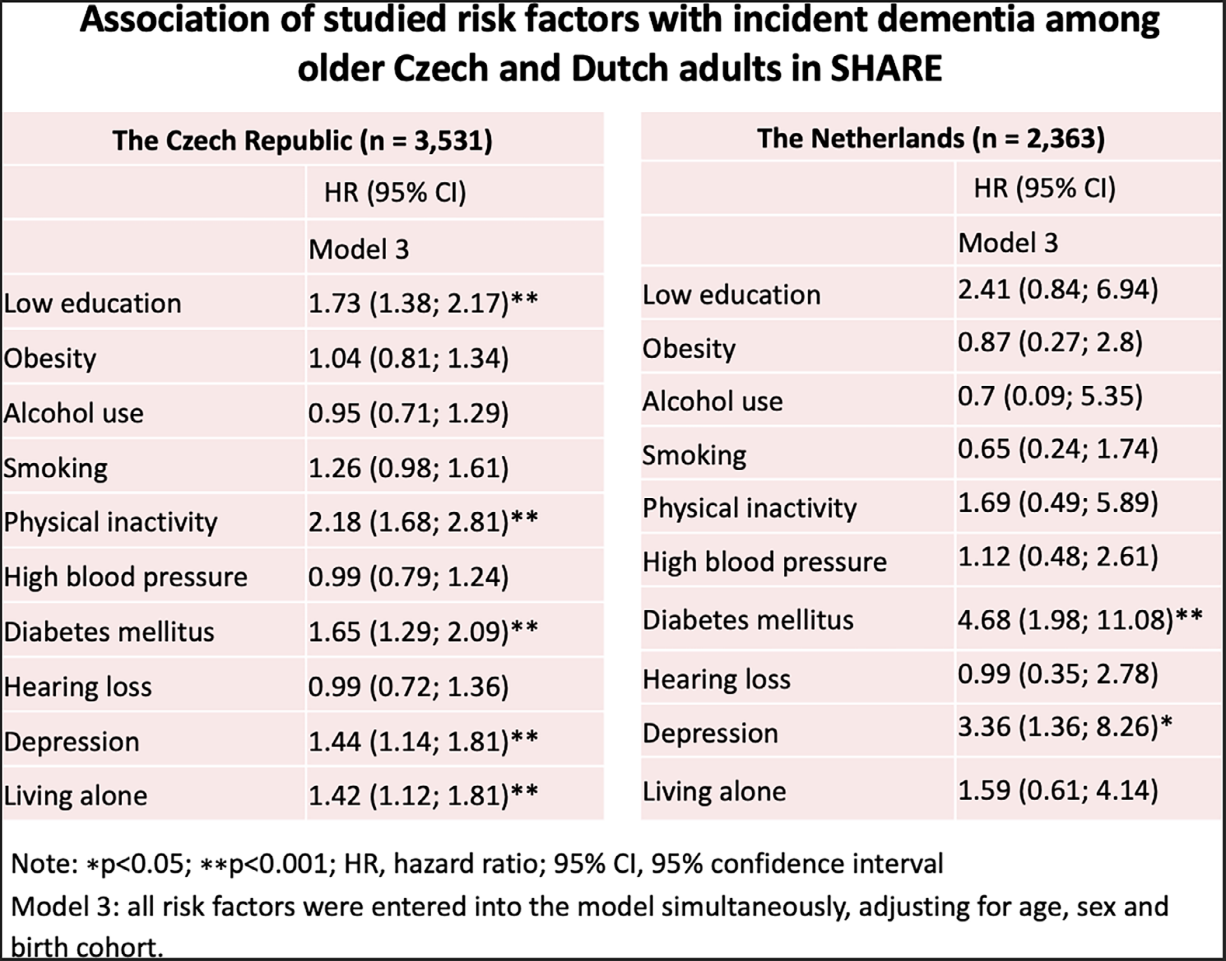# The role of risk factors for development of dementia in Czechia and in the Netherlands

**DOI:** 10.1002/alz70860_102185

**Published:** 2025-12-23

**Authors:** Matěj Kučera, Pavla Brennan Kearns, Dominika Dominika James, Hana M. Broulíková, Jitsa Soepnel

**Affiliations:** ^1^ Vrije Universiteit Amsterdam, Amsterdam, Netherlands; ^2^ 2nd Medical School, Charles University, Prague, Czech Republic; ^3^ The National Institute of Mental Health, Klecany, Czech Republic; ^4^ 2nd Medical School, Charles University, Prague, Czech Republic, Czech Republic; ^5^ Second Medical Faculty Charles University, Prague, Czech Republic; ^6^ Department of Health Sciences, Faculty of Science, Vrije Universiteit Amsterdam, Amsterdam, Netherlands; ^7^ Alzheimer Center Amsterdam, Neurology, Vrije Universiteit Amsterdam, Amsterdam UMC location VUmc, Amsterdam, Netherlands

## Abstract

**Background:**

The burden of risk factors for dementia is greater in Czechia, a country situated in Central and Eastern Europe, than in the Netherlands, a country in Western Europe. It is not known if the impact of the risk factors on the risk of dementia differs for these two countries. This study aims to investigate differences in the associations of ten established risk factors for dementia between populations in Czechia and the Netherlands.

**Method:**

We studied individuals older than 65 years participating in at least two waves of the SHARE residing in Czechia (58% females, 70 mean age) and in the Netherlands (51.4% females, 70.7 mean) Data on the ten risk factors were collected using computer‐assisted personal interviewing. Incident dementia was defined using an adapted Lang‐Weir classification based on scores in immediate recall, delayed recall, and instrumental activities of daily living. Cox regression was applied to estimate the hazard ratio (HR) with 95% confidence interval (CI) for the association between the risk factors and incident dementia, adjusting for age, sex, and birth cohort in three models.

**Result:**

In Czechia, low education, physical inactivity, diabetes mellitus, depression and living alone were associated with the incident dementia, when analyzed in separate models and adjusted for age, sex, and birth cohort in Model 1. The associations remained when these five exposures were entered into one model (Model 2) as well as when all ten risk factors were entered at the same time (Model 3: low education HR 1.7, 95% CI 1.4‐2.2; physical inactivity HR 2.2, 95% CI 1.7‐2.8; diabetes mellitus HR 1.7, 95% CI 1.3‐2.1; depression HR 1.4, 95% CI 1.1‐1.8; living alone HR 1.4, 95% CI 1.1‐1.8). In the Netherlands same factors, except for living alone were associated with a greater hazard of dementia in Model 1, but only diabetes mellitus and depression remained associated in further models (Model 3: diabetes mellitus HR 4.7, 95% CI 2.0‐11.1; depression HR 3.4, 95% CI 1.4‐8.3).

**Conclusion:**

The impact of risk factors on the risk of dementia may differ across countries, emphasizing the importance of tailoring prevention strategies to specific national contexts based on specific risk factor profiles.